# Transcriptome profiling of *longissimus lumborum* in Holstein bulls and steers with different beef qualities

**DOI:** 10.1371/journal.pone.0235218

**Published:** 2020-06-25

**Authors:** Yan Li, Meimei Wang, Qiufeng Li, Yanxia Gao, Qian Li, Jianguo Li, Yufeng Cao

**Affiliations:** 1 College of Veterinary Medicine, Hebei Agricultural University, Baoding, Hebei, China; 2 College of Animal Science and Technology, Hebei Agricultural University, Baoding, Hebei, China; 3 Hebei Animal Husbandry and Veterinary Institute, Baoding, Hebei, China; Universidade Federal de Viçosa, BRAZIL

## Abstract

Previous research regarding Holstein cows has mainly focused on increasing milk yield. However, in order to maximize the economical profits of Holstein cattle farming, it is necessary to fully take advantage of Holstein bulls to produce high-grade beef. The present study aims to investigate different transcriptomic profiling of Holstein bulls and steers, via high-throughput RNA-sequencing (RNA-seq). The growth and beef quality traits of Holstein steers and bulls were characterized via assessment of weight, rib eye area, marbling score, shear force and intramuscular fat percentage of the *longissimus lumborum* (LL) muscle. The results indicated that castration improved the meat quality, yet reduced the meat yield. Subsequently, RNA-seq of the LL muscle from Holstein steers and bulls revealed a total of 56 differentially expressed genes (DEGs). We performed the functional enrichment analysis in Gene Ontology (GO) annotations of the DEGs using GOseq R package software and Kyoto Encyclopedia of Genes and Genomes (KEGG) pathway analysis using KOBAS tool. Through the integrated analysis of DEGs with reported QTLs and SNPs, seven promising candidate genes potentially affecting the beef quality of LL muscle following castration were discovered, including muscle structural protein coding genes (*MYH1*, *MYH4*, *MYH10*) and functional protein coding genes (*GADL1*, *CYP2R1*, *EEPD1*, *SHISA3*). Among them, *MYH10*, *GADL1*, *CYP2R1*, *EEPD1* and *SHISA3* were novel candidate genes associated with beef quality traits. Notably, *EEPD1* was associated with both meat quality and reproduction traits, thus indicating its overlapping role in responding to hormone change, and subsequently inducing beef quality improvement. Our findings provide a complete dataset of gene expression profile of LL in Holstein bulls and steers, and will aid in understanding how castration influence meat yield and quality.

## Introduction

Holstein cattle, a common cattle breed, has been mainly raised for dairy purpose. However, to meet the growing demand for beef, more and more Holstein cattle are also used to produce meat. For many decades, research studies have focused on increasing milk yield and milk quality of Holstein cows, but have ignored the utilization value of Holstein bulls in beef production [1–3]. Therefore, in order to maximize economical profits of Holstein cattle farming, it is necessary to take fully advantage of Holstein bulls to produce high-grade beef. The beef yield and quality can be influenced by many factors, such as breed, feeding, age and castration [4–6]. Castration is an effective way to manage Holstein bulls, by which remarkably resulting in higher quality beef in steers [7].

Beef quality may be assessed by intramuscular fat (IMF) deposition, as well as by marbling and fatty acid composition, which further determines meat palatability, including flavor, tenderness and juiciness [8–10]. Numerous trials regarding beef cattle breeds such as *Bos indicus* bulls, Qinchuan cattle, Chinese Simmental cattle and Korean cattle, have suggested that castration reduced meat yield, yet improved meat quality, including fatty acid composition and IMF content [11–14]. Two recent studies physicochemically characterized the beef quality of Holstein bulls, and the results suggested that the steer beef contained higher redness, intramuscular fat and soluble collagen, thus indicating a better eating quality than bull beef [15,16].

The gene expression profiling changes of the *longissimus dorsi* in beef cattle resulting from castration were described by two research groups. Zhou *et al*. identified several differentially expressed genes, such as *ACTIN*, *TPM2*, *IGF1* and *LIPE*, between Chinese Simental steers and bulls, by means of the suppressive subtractive hybridization method, and the results indicated that these genes may contribute to the regulation of steer beef quality [12]. Jeong *et al*. profiled the transcriptomic changes of LL muscle of Korean cattle following castration using microarray. They discovered several lipid metabolism genes differentially expressed in steers and bulls, such as *PLIN2*, *ATP6V1C1* and *COX11*, thus indicating that the improvement of beef quality by castration attributed to better IMF deposition in *longissimus dorsi* muscle [17]. With the advantages of high-throughput sequencing technologies, the genetic mapping of quantitative trait loci (QTL) and genome wide association studies have generated well-defined genetic maps for carcass and meat quality traits [18–22]. In addition, RNA-seq of beef cattle groups with divergent meat quality has revealed several meat quality related metabolic pathways and genes, such as PPAR signaling genes, cell morphology genes, lipid metabolism genes, and adipocytokine signaling pathway [23–25]. However, RNA-seq technology has yet to be used to profile the transcriptomic changes of bull muscle followed by castration. Therefore, the characterizations of castration effects on the gene expression profiles of Holstein bulls using high-throughput RNA-seq can provide valuable molecular and genetic information regarding how castration improves beef quality. The aim of the present study is to identify the important genes that respond to castration, and subsequently aid in improving beef quality in Holstein bulls via RNA-seq.

## Materials & methods

### Animals and management

The animal experiment performed in this study was approved by the Animal Ethical and Welfare Committee (AEWC) at the College of Animal Science and Technology of Hebei Agricultural University (Approval number: 17–06). The animals were treated in accordance with the China National Institute of Health guidelines. Twenty healthy 11-month-old Holstein bulls with similar live weight, from different families, were selected at the cattle farm of Fucheng Wufeng Food Co., Ltd. (Langfang, China). The average live weight was 271.25±13.73 kg in this population and they were randomly assigned to one of two groups. Bulls of one group were castrated by surgically removing the testicles, while the other group had their testicles remain intact. All the animals were housed in the open barn at the cattle farm of Fucheng Wufeng Food Co., Ltd. Feeding management of the two groups was kept consistent before selection and throughout the experimental period. Referring to Nutrition Requirement of Beef Cattle (2000) and Japanese Feeding Standard for Beef Cattle (2008), the basic diet formulation was adjusted along with the growth of body weight (S1 Table). The dietary nutrition levels of the two groups were identical. Following a 7 d adaption period, the experiment lasted for a total of 448 d.

### Slaughtering, tissue and blood sampling

After 448 d of feeding, all of the cattle were driven to the abattoir (Fucheng Wufeng Food Co., Ltd., Langfang, China), located next to the feedlot. The cattle were feed fasted for 24 h and water fasted for 3 h before slaughter. Following electric shock, all cattle were slaughtered by exsanguination. The slaughtering process was performed according to the operating procedure of cattle slaughtering outlined in the China National Standard. Meanwhile, the hot carcass weight was recorded. Next, the carcasses were divided into two parts, weighed and chilled at 0~4°C for 48 h. After chilling, 1 kg of LL muscle on the right carcass side between the 12th and 13th ribs was removed. Half of the muscle samples were stored at -20°C for beef quality characterization, while the other half was cut into several pieces and placed quickly in liquid nitrogen, followed by storage at -80 °C until analysis.

### Measurements of beef quality parameters

All of the measurements below were performed on the -20°C stored muscle samples. The perimeter of the rib eye area was traced on a sheet of sulfuric paper, followed by calculation with a planimeter (Jilin University, China). The marbling grade was determined according to the Japanese Marbling scores (5-Excellent; 4-Good; 3-Average; 2-Below average; 1-Poor). Immediately after this, the meat sample was boiled and cooled, and the shear force was measured using a Warner-Bratzler shear force machine (Bodine Electric Co., Chicago, IL, USA) according to the manufacturer’s instructions. According to the AOAC official methods, the meat samples were dried in a freeze dryer (LaboGene, Allerod, Denmark) to determine the contents of H_2_O, crude protein and IMF. The Kjeldahl method was then applied to analyze the crude protein content using a Kjeltec 8400 Analyzer Unit (Foss Analytical, Höganäs, Sweden). Finally, the total IMF percentage was determined by the Soxhlet extraction method.

### RNA extraction and quality analysis

Three cattle were randomly selected from both the bull and steer groups for RNA-sequencing analysis. The total RNA was extracted from the tissues using TRIzol Reagent (Life Technologies, CA, USA) according to the manufacturer’s instructions. A Qubit^®^ RNA Assay Kit (Invitrogen, CA, USA) was used to determine the RNA concentration with a Qubit^®^ 2.0 Flurometer (Life Technologies, CA, USA). The RNA degradation and contamination were monitored by 1% agarose gel. The RNA purity was measured by a NanoPhotometer^®^ spectrophotometer (IMPLEN, CA, USA). Finally, the RNA integrity was checked using a Bioanalyzer 2100 RNA 6000 Nano Kit (Agilent Technologies, CA, USA).

### Library preparation and RNA sequencing

The RNA sequencing libraries were constructed according to the manufacturer’s instructions of the NEBNext^®^ Ultra^™^ RNA Library Prep Kit for Illumina^®^ (NEB, MA, USA). Index codes were added, so as to attribute sequences to each sample. Next, mRNA was isolated using poly-T oligo-attached magnetic beads and fragmented by divalent cations in a NEB Next First Strand Synthesis Reaction Buffer (5X) under increased temperature. Subsequently, the first and second strand cDNA were synthesized using random hexamer primer. The 3’ ends of DNA fragments were adenylated, followed by ligation with a NEBNext Adaptor. cDNA fragments of 150~200 bp were selected with an AMPure XP system (Beckman Coulter, CA, USA). PCR was performed with Universal PCR primers and an Index (X) Primer. The library quality was checked by an Agilent Bioanalyzer 2100 system. TruSeq PE Cluster Kit v3-cBot-HS (Illumina, CA, USA) was used for cluster generation. The final RNA-seq libraries were constructed using an Illumina Hiseq 2500 platform (Illumina, CA, USA), which generated 125 bp/150 bp paired-end reads.

### Sequencing data analysis

In order to obtain clean data, raw data (fastq format) were processed through in-house perl scripts. In this step, clean data were obtained by removing reads containing adapters, low quality reads (the proportion of the read bases with Phred quality score≤20 is over 50% of the reads), and reads with proportion of N greater than 10%. Subsequently, the Q20 (the percentage of read bases with Phred quality score >20), Q30 (the percentage of read bases with Phred quality score >30), and GC contents of the clean data were calculated. The downstream study analyzed the clean data with high quality. The paired-end clean reads were mapped to the reference genome of Bos taurus UMD 3.1.1 (https://www.ncbi.nlm.nih.gov/genome/82?genome_assembly_id=214974) using TopHat v2.0.12. The index of the reference genome was built using Bowtie v2.2.3.

### Gene expression quantification and differential expression analysis

The gene expression level was estimated as fragments per kilobase of transcript per millions fragments mapped reads (FPKM), which was calculated based on the length of the gene and reads count mapped to this gene. HTSeq v0.6.1 was utilized for counting the read numbers mapped to each gene. Differential expression analysis of Holstein bulls and steers was performed using the DESeq R package (1.18.0). Benjamini and Hochberg’s approach was applied to correct the resulting P-values to control the false discovery rate. Genes with a corrected P-value <0.05 were considered as differentially expressed genes (DEGs).

### Gene Ontology (GO) and pathway enrichment analysis of DEGs

Gene Ontology (GO) of DEGs was implemented using the GOseq R package. KOBAS software was applied to calculate the statistical enrichment of DEGs in the KEGG pathways (http://www.genome.jp/kegg/).

### QTL-SNP screening analysis of DEGs

To associate the DEGs with beef quality traits, a QTL-SNP screening analysis was performed to determine the candidate genes [26,27]. Firstly, the DEGs with the physical position located within the region of reported QTLs related with beef quality traits (https://www.animalgenome.org/cgi-bin/QTLdb/BT/index) were selected. The genetic distance between the DEGs and QTL peak was calculated. Secondly, we created a pool of SNPs related with beef quality traits reported by previous GWAS. Physical position of the DEGs and the reported SNPs were compared, and the DEGs with distance to significant SNPs less than 5 Mb were selected. Finally, combining the QTL and SNP screening results, the overlapped DEGs those were both located inside of QTLs and near significant SNPs were selected as candidate genes associated with beef quality traits.

### qRT-PCR

To confirm the sequencing results, nine DEGs were randomly selected for qRT-PCR. The total RNA (1 μg) was reverse transcribed into first strand cDNA by a Quantitect^®^ reverse transcription kit (Qiagen, Hilden, Germany) according to the manufacturer’s instructions. Primers for qPCR were designed using the primer-BLAST tool on the NCBI website (S2 Table). The transcript levels of the tested genes were normalized to β-actin and GAPDH in qRT-PCR, and calculated using the 2^-△△ct^ method. The qPCR reactions were performed in triplicates using iQ SYBR Green Supermix (BioRad, CA, USA). The amplified conditions were as follows: 95°C for 3 min; 40 cycles of 95°C for 15 s, 60°C for 15 s and 72°C for 20 s; 72°C for 10 min.

### Statistical analysis

The data of the growth and beef quality characteristics from the steers’ group and the bulls’ group obeyed normal distribution with the Kolmogorov-Smirnov test, followed by analysis with the Independent-Sample T test using SPSS 19.0 software. The results were shown as mean value ± standard error. A probability of P ≤0.05 was considered as significant, while P≤0.01 was highly significant, and 0.05<P<0.1 was trendy. Pearson’s correlations between transcript abundance from RNA-seq and qRT-PCR were analyzed using Minitab18 statistical software.

## Results

### Growth and beef quality characteristics of Holstein bulls and steers

The growth and beef qualities characteristics of the bulls and steers are listed in Table 1. Castration negatively affected the carcass weight of the steers (P<0.05). In comparison with the bulls, the steers showed significantly decreased rib eye area (p<0.01), thus it can be concluded that castration resulted in slower growth and lower meat yield. However, the steers showed superior marbling (*P*<0.05) and higher IMF content (*P*<0.01) of LL muscle than the bulls. At the same time, the LL of the steers showed slightly lower shear force than the bulls, indicating that the LL of the steers possessed superior tenderness. Therefore, it was concluded that the castration significantly improved steer beef quality, and the LL samples could be used for RNA-seq to detect genes associated with beef quality traits.

**Table 1:** Effect of castration on growth and beef quality of *longissimus lumborum* in Holstein bulls.

	Bulls	Steers	SEM	P-value
**Initial weight (kg)**	275.90±5.62a	269.10±3.18a	6.458	0.306
**Carcass weight (kg)**	455.40±7.82a	434.50±5.88b	9.784	0.047
**Rib eye area (cm**^**2**^**)**	91.00±2.74a	76.10±2.93b	4.012	0.002
**Marbling score**	2.30±0.15b	3.10±0.22a	0.269	0.008
**Shear force**	3.62±0.56a	3.57±0.51a	0.760	0.949
**Water (%)**	72.67±1.14a	67.49±1.48b	1.866	0.024
**IMF (%)**	7.04±0.69b	12.29±1.90a	2.02	0.048
**Protein (%)**	20.59±0.40a	19.53±0.41a	0.573	0.102

### RNA sequencing of bovine LL muscle

Next, a total of 305.57 million clean reads, with an average of 50.92 million for each sample (ranging from 47.80 to 54.43 million) were generated (S3 Table). The respective quality values of Q20 and Q30 were 95.29% and 88.84%. In this study, 88.32% of the total reads which mapped uniquely to the reported *Bos taurus* genome (http://www.ncbi.nlm.nih.gov/genome/guide/cow/index.html) were analyzed (S4 Table). Reads which could not be mapped (8.75%), or mapped to multiple positions (2.93%), were excluded from the following analysis (S4 Table). Through FPKM calculation, a total of 11,521 and 11,511 expressed genes were respectively detected in the bulls and steers. Among the expressed genes, a total of 10437 genes were common for the bulls and steers. In order to better characterize the difference of gene expression intensities, the FPKM values of gene expression were divided into five expression levels, from lowest to highest (S5 Table).

### Differentially expressed genes (DEGs)

**Fig 1:**
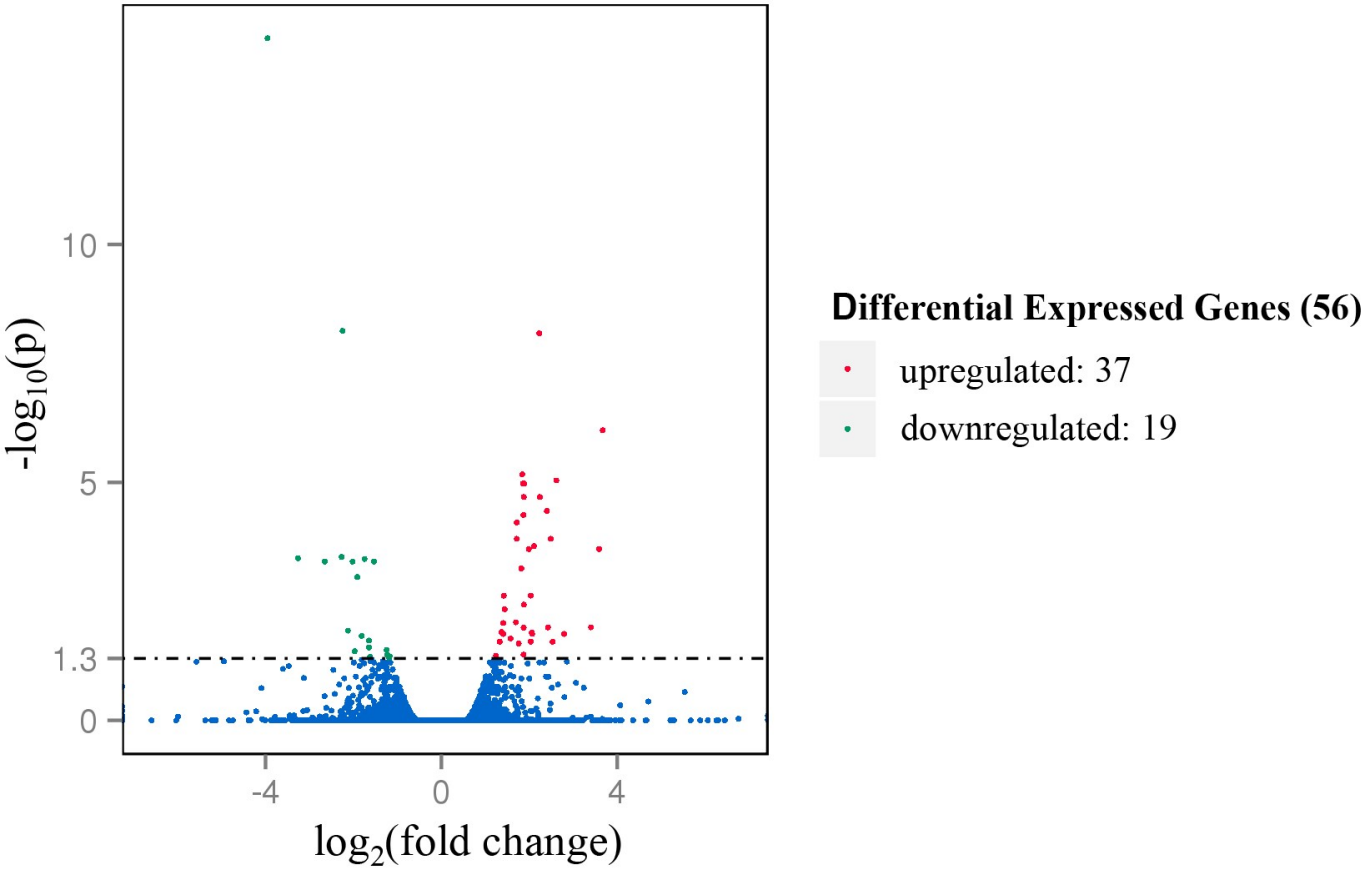
Volcano Plot of DEGs in *LL* muscle comparing steers versus bulls. The x-axis shows the values of log_2_ (fold change), while the average expression values of log_10_ (p) are displayed by the y-axis. The red and green dots represent the significantly differentially expressed transcripts (p < 0.05) comparing the *LL* muscle of steers versus bulls, with red for upregulated genes and green for downregulated genes. The blue dots indicate the transcripts with expression levels which are not statistically significant (p > 0.05) comparing steers and bulls.

In order to characterize how the gene expression profiles of the LL muscle were impacted by castration, we compared the gene expression levels of the steers versus the bulls using DESeq method. A total of 56 significant DEGs were identified, among which 37 were upregulated and 19 were downregulated in the steers. Among these, 44 DEGs were known transcripts, of which 40 were transcripts of annotated genes and 4 were transcripts of pseudogenes. Twelve novel transcripts were detected, with 10 upregulated and 2 downregulated. The transcription profiles of the bulls and steers are shown in Fig 1. All of the annotated DEGs identified from comparison between the LL muscle of steers and bulls are listed in Table 2.

**Table 2:** Forty annotated differentially expressed genes in *LL muscle* identified between Holstein steers and bulls.

*Gene ID*	*Official Full Name*	*Gene Symbol*	*Chr*	*Position*	*log*_*2*_ *Fold Change (steers/bulls)*	*P-Value*
**ENSBTAG00000000448**	3-hydroxybutyrate dehydrogenase 1	*BDH1*	1	72572941–72608810	1.8192	6.45E-04
**ENSBTAG00000002151**	ankyrin repeat and SOCS box containing 15	*ASB15*	4	88697392–88729365	1.7126	6.98E-05
**ENSBTAG00000002527**	zinc finger SWIM-type containing 4	*ZSWIM4*	7	12959854–12977638	-1.7474	4.06E-04
**ENSBTAG00000003403**	peptidyl arginine deiminase 2	*PADI2*	2	136049644–136103406	1.88	1.06E-05
**ENSBTAG00000005847**	Rho associated coiled-coil containing protein kinase 2	*ROCK2*	11	86501577–86583652	-1.534	4.61E-04
**ENSBTAG00000005857**	solute carrier family 6 member 1	*SLC6A1*	22	55695783–55714644	1.716	1.53E-04
**ENSBTAG00000006754**	D-box binding PAR bZIP transcription factor	*DBP*	18	55722336–55729578	1.4405	4.62E-03
**ENSBTAG00000007062**	insulin like growth factor binding protein 5	*IGFBP5*	2	105378991–105397646	2.7936	1.53E-02
**ENSBTAG00000007131**	glutamate decarboxylase like 1	*GADL1*	22	5258463–5452369	1.7586	2.44E-02
**ENSBTAG00000007635**	phospholipase C like 1	*PLCL1*	2	86718341–87086748	1.4057	9.02E-03
**ENSBTAG00000008103**	aldehyde dehydrogenase 1 family member A1	*ALDH1A1*	8	49354207–49408166	1.3273	2.22E-02
**ENSBTAG00000008353**	cyclin dependent kinase inhibitor 1A	*CDKN1A*	23	10560499–10568780	-2.2471	6.56E-09
**ENSBTAG00000008807**	F-box and leucine rich repeat protein 22	*FBXL22*	10	46461587–46466076	1.8751	3.71E-03
**ENSBTAG00000008866**	glucosidase alpha, neutral C	*GANC*	10	37754445–37817713	1.8652	4.84E-05
**ENSBTAG00000008940**	neuronal pentraxin 1	*NPTX1*	19	52679104–52685498	-2.0194	4.61E-04
**ENSBTAG00000009148**	glycoprotein A33	*GPA33*	3	1726504–1779667	-2.1275	1.30E-02
**ENSBTAG00000010389**	starch binding domain 1	*STBD1*	6	92967767–92971371	2.2268	7.38E-09
**ENSBTAG00000010419**	cytochrome P450 family 2 subfamily R member 1	*CYP2R1*	15	38420926–38445583	-1.913	9.68E-04
**ENSBTAG00000011381**	solute carrier family 30 member 3	*SLC30A3*	11	72364682–72373190	-2.2679	3.70E-04
**ENSBTAG00000011548**	adenosine monophosphate deaminase 1	*AMPD1*	3	28756908–28768496	1.8424	6.75E-06
**ENSBTAG00000011808**	Growth/differentiation factor 8	*MSTN*	2	6213566–6220196	2.034	2.22E-02
**ENSBTAG00000046587**	cystatin E/M	*CST6*	29	44766936–44768173	1.8718	1.13E-02
**ENSBTAG00000015018**	fibronectin type III and SPRY domain-containing protein 2	*FSD2*	21	23501906–23537791	1.4181	2.39E-03
**ENSBTAG00000015402**	protein GREB1	*GREB1*	11	86199420–86268193	-3.9542	4.65E-15
**ENSBTAG00000016269**	malic enzyme 2	*ME2*	24	50870262–50928290	-1.622	4.65E-02
**ENSBTAG00000016444**	reticulophagy regulator 1	*RETREG1*	20	56709603–56758641	-1.2271	4.16E-02
**ENSBTAG00000017765**	glutathione S-transferase M2	*GSTM2*	3	33824401–33834874	1.6982	8.71E-03
**ENSBTAG00000016591**	RAB11 family interacting protein 3	*RAB11FIP3*	25	404964–464143	-1.1768	4.52E-02
**ENSBTAG00000016676**	phosphotriesterase related	*PTER*	13	31202455–31278293	1.3723	1.40E-02
**ENSBTAG00000018088**	SET binding protein 1	*SETBP1*	24	45025456–45141793	1.4051	1.53E-02
**ENSBTAG00000018204**	myosin heavy chain 1	*MYH1*	19	30110728–30134757	1.988	2.52E-04
**ENSBTAG00000019065**	endonuclease/exonuclease/phosphatase family domain containing 1	*EEPD1*	4	61354648–61476979	1.2436	4.40E-02
**ENSBTAG00000019954**	abhydrolase domain containing 2	*ABHD2*	21	20998719–21106763	-1.6449	2.11E-02
**ENSBTAG00000021151**	myosin heavy chain 10	*MYH10*	19	28680825–28800880	-1.1847	4.97E-02
**ENSBTAG00000034411**	shisa family member 3	*SHISA3*	6	62877287–62880849	-1.9731	3.54E-02
**ENSBTAG00000037794**	myosin heavy chain 4	*MYH4*	19	30080604–30103436	2.2376	2.03E-05
**ENSBTAG00000038584**	olfactomedin 1	*OLFM1*	11	106675880–106712955	1.5765	1.91E-02
**ENSBTAG00000039574**	nephrocan	*NEPN*	9	33595874–33619430	2.4845	1.53E-04
**ENSBTAG00000040126**	desmoglein 4	*DSG4*	24	26038529–26078704	-2.6521	4.61E-04
**ENSBTAG00000040398**	KIAA1211 ortholog	*KIAA1211*	6	73368358–73396166	2.0568	1.44E-02

### Validation of RNA-seq by qRT-PCR

**Fig 2:**
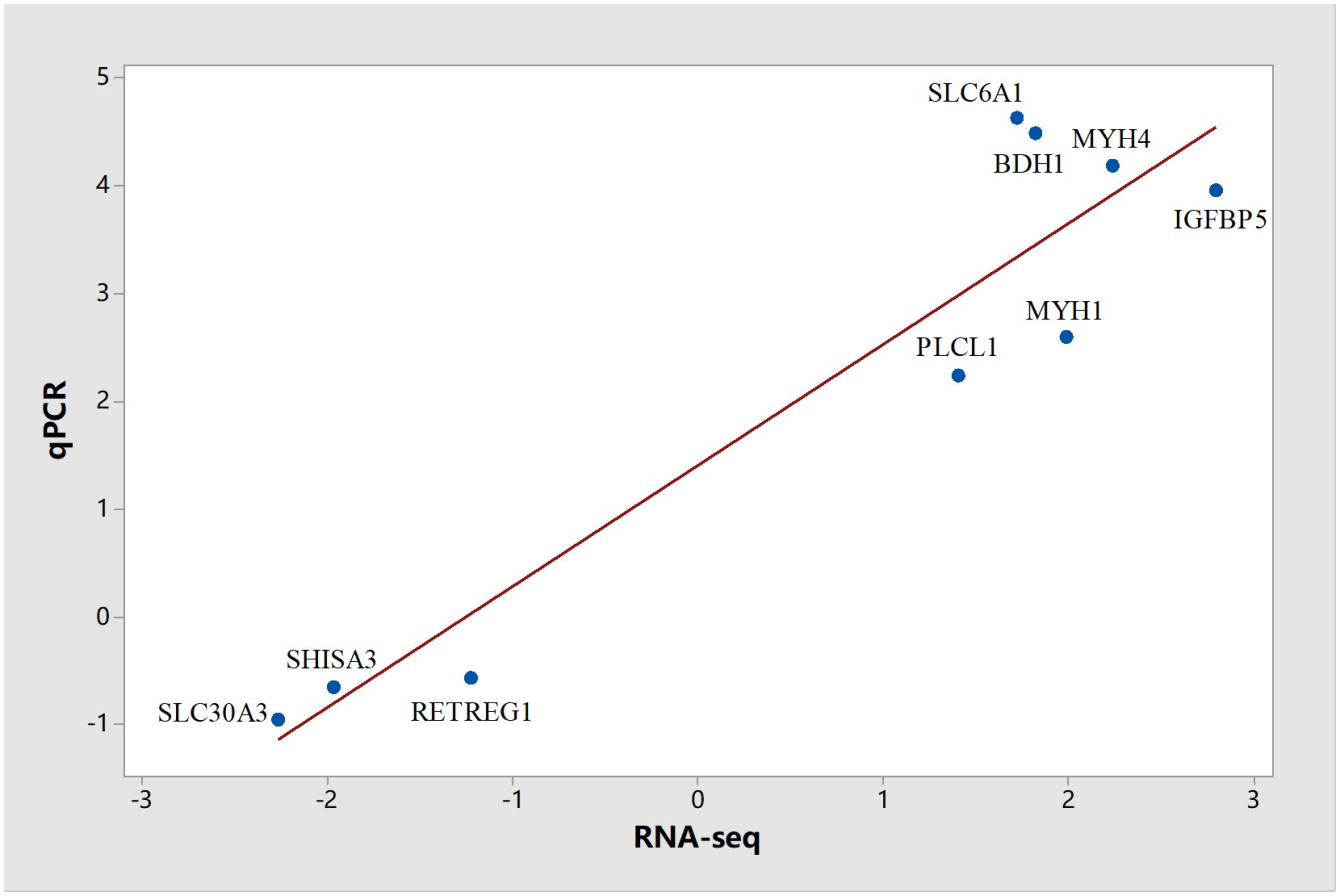
Correlations of mRNA expression levels of 9 random DEGs in *LL* muscle of steers versus bulls by qRT-PCR and RNA-seq. The x-axis indicates the log_2_ (ratio of mRNA levels) using RNA-seq, and the y-axis displays the log_2_ (ratio of mRNA level) measured by qRT-PCR. The blue dots represent the tested genes. The red line indicates the scatterplot of qPCR vs RNA seq.

In order to verify the RNA-seq results, we randomly selected 9 genes from the 41 annotated DEGs for qRT-PCR, namely *IGFBP5*, *MYH1*, *PLCL1*, *SLC6A1*, *BDH1*, *MYH4*, *SLC30A3*, *PETREG1* and *ME2*. The comparisons of transcript abundance detected by qRT-PCR and RNA-Seq are illustrated in Fig 2, showing the correlated gene expression levels using these two approaches. Consequently, it was shown that the RNA-seq data of this study are reproducible and convincible.

### GO and pathway analysis of the DEGs

**Fig 3:**
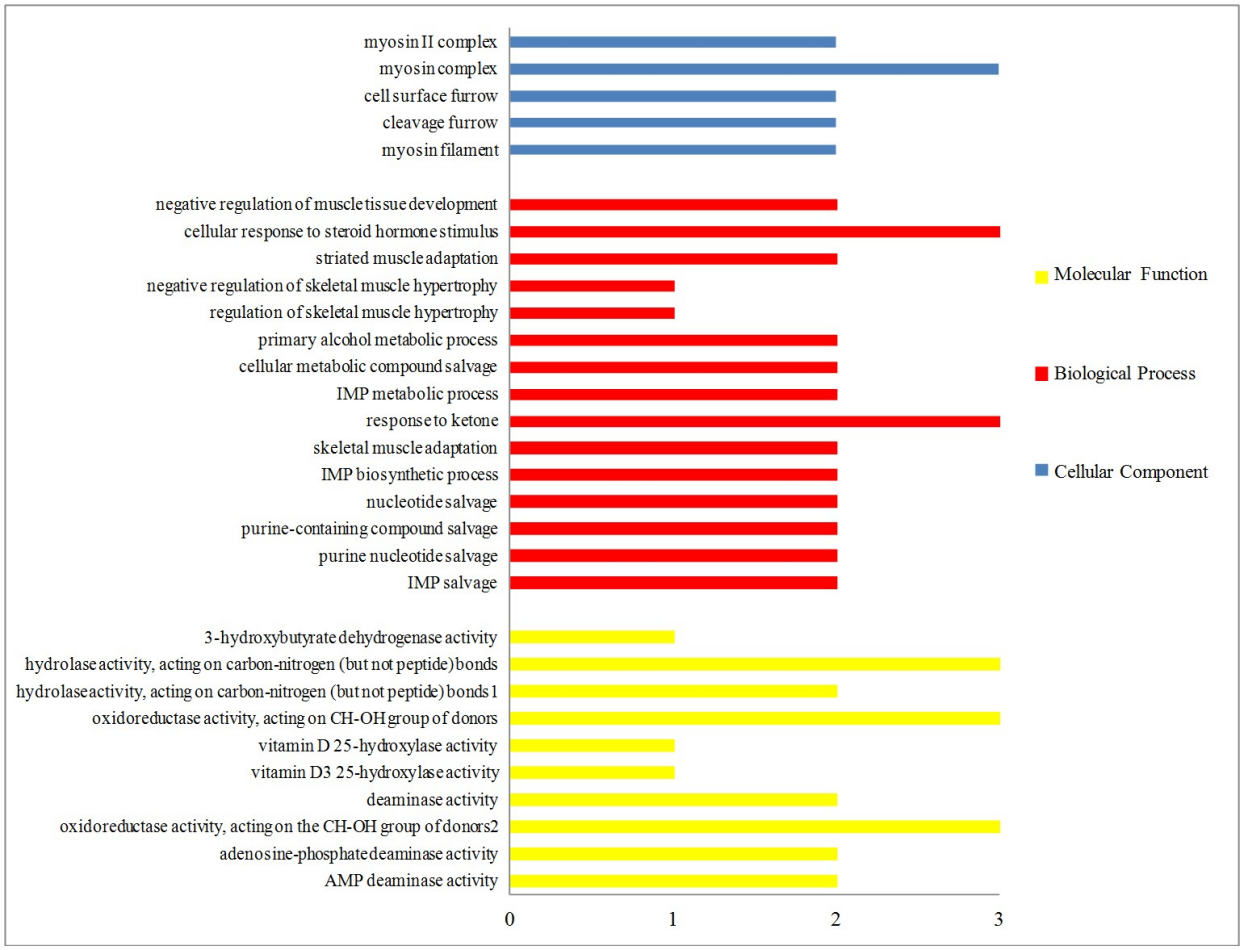
Most highly enriched GO terms of DEGs between Holstein steers and bulls. The x-axis displays the number of DEGs, and the y-axis represents the GO terms. The bar colors correspond to different GO categories, with yellow for molecular function, red for biological process and blue for cellular component. ^1^Hydrolase activity, acting on carbon-nitrogen (but not peptide) bonds, in cyclic amidines. ^2^Oxidoreductase activity, acting on the CH-OH group of donors, NAD or NADP as acceptor.

In order to further investigate the physiological characteristics that were affected by castration in the LL muscle, 56 DEGs were analyzed for their functions via Gene Ontology (GO). Most of the GO categories were associated with muscle development, cell division, various enzymatic activities and nucleotide metabolism (Fig 3). Specifically, the most enriched cellular components were myosin filament, cleavage furrow, cell surface furrow and myosin complex, most of which relate to muscle growth and development. The most highly enriched molecular functions include deaminase activities, oxidoreductase activities, vitamin D_3_ 25-hydroxylase activity and hydrolase activities, all of which either directly or indirectly participated in fatty acid metabolism. An additional GO term clearly associated with fatty acid synthesis in the top 30 enriched categories is a response to ketones. Meanwhile, the top biological processes consist of IMP salvage, purine nucleotide salvage, purine-containing compound salvage and nucleotide salvage, which were all related with the purine nucleotide cycle, which is the ultimate function in muscle energy production.

To discover the metabolic pathways associated with beef quality, we performed metabolic pathway analysis on the DEGs. The details of the top 12 enriched pathways in the LL muscle by comparing bulls and steers are listed in Table 3. The pathways which were related with fatty acids metabolism were synthesis and degradation of ketone bodies, butanoate metabolism, purine metabolism, galactose metabolism, linoleic acid metabolism, and pyruvate metabolism. Other pathways were involved in muscle cell growth, such as tight junction, oxytocin signaling pathway and proteoglycans in cancer.

**Table 3:** Most highly enriched KEGG pathways of DEGs between Holstein steers and bulls.

*KEGG Terms*	*Input number*	*P-Value*	*Gene name*
Tight junction (cell polarity; muscle cell development)	3	0.006	MYH4, MYH1, MYH10
GABAergic synapse	2	0.025	SLC6A1, PLCL1
Synthesis and degradation of ketone bodies (ketone bodies degraded to acetyl-CoA for fatty acids synthesis)	1	0.032	BDH1
Oxytocin signaling pathway (cell proliferation; contraction)	2	0.067	ROCK2, CDKN1A
Butanoate metabolism (Acetyl-CoA for fatty acids synthesis)	1	0.076	BDH1
Purine metabolism	2	0.086	ENSBTAG00000045889 (pseudogene), AMPD1
Galactose metabolism (glycerol for fatty acids synthesis)	1	0.087	GANC
Linoleic acid metabolism (fatty acid metabolism)	1	0.094	CYP2R1
Pyruvate metabolism (linked to fatty acid biosynthesis through Malonyl-CoA	1	0.101	ME2
Bladder cancer (X)	1	0.101	CDKN1A
Proteoglycans in cancer (cell growth)	2	0.107	ROCK2, CDKN1A
Metabolic pathways	6	0.125	ALDH1A1, AMPD1, ENSBTAG00000045889 (pseudogene), GANC, CYP2R1, BDH1

### Candidate genes associated with beef quality traits by QTL-SNP screening

In order to further screen the DEGs for the candidate genes related with beef quality traits, we analyzed the DEGs in the animal QTL database (https://www.animalgenome.org/cgi-bin/QTLdb/index). By comparing the gene position of the DEGs on the chromosome with the QTLs region, 9 out of 40 annotated DEGs were discovered (S6 Table). In addition, we created a genome-wide association studies (GWAS) database by pooling the published SNPs related with meat quality traits for further analysis. The differentially expressed genes are considered as related to the specific SNP associated traits only if the position of the gene on the chromosome is at a distance of less than 5 Mb from that of the SNP (S7 Table). A total of 39 out of 40 annotated DEGs were found as potential genes related to beef quality. Taken together, seven overlapping DEGs, namely *GADL1*, *CYP2R1*, *MYH1*, *EEPD1*, *MYH10*, *SHISA3* and *MYH4*, are considered as promising candidate genes responsible for the differences in LL muscle qualities between Holstein bulls and steers (Table 4).

**Table 4:** Candidate DEGs associated with meat quality traits.

*Gene Information*			*Reported QTLs*				*Reported SNPs*			
Gene Symbol	Chr^1^	Position (bp)^2^	QTL ID^3^	Distance to QTL peak (cM)	Traits^4^	Ref^5^	SNP Name	Distance to SNP (Mb)	Traits^4^	Ref^5^
*EEPD1*	4	61354648–61476979	20355	0.6	CW	[28]	Hapmap42645-BTA-70875	1.84	CC	[29]
			20368	0.6	FT12R	[28]				
*MYH10*	19	28680825–28800880	22873	-22.3	IMF	[30]	UA-IFASA-9813	3.92	CW	[29]
							ARS-BFGL-NGS-88422	0.82	CC	[29]
			22872	-2.6	IMF	[30]				
*CYP2R1*	15	38420926–38445583	24706	-6.7	LDMA	[31]	ARS-BFGL-NGS-117790	2.12	CC	[29]
*GADL1*	22	5258463–5452369	37164	-2.0	LMY	[29]	ARS-BFGL-NGS-27027	0.06	CC	[29]
*MYH1*	19	30110728–30134757	22873	-19.9	IMF	[30]	UA-IFASA-9813	2.58	CW	[29]
							ARS-BFGL-NGS-88422	2.25	CC	[29]
*SHISA3*	6	62877287–62880849	20764	-0.8	SF	[33]	BTA-76543-no-rs	1.13	IMFP	[32]
*MYH4*	19	30080604–30103436	22873	-19.9	IMF	[30]	UA-IFASA-9813	2.61	CW	[29]
							ARS-BFGL-NGS-88422	2.22	CC	[29]

^1^ Chromosome in *B*. *taurus*.

^2^ Gene position on the UMD3.1.1 bovine genome assembly.

^3^ QTL information retrieved on the Animal Quantitative Trait Loci (QTL) Database (Animal QTLdb) (https://www.animalgenome.org/cgi-bin/QTLdb/index).

^4^ CW: carcass weight; IMF: intramuscular fat; FT12R: fat thickness at the 12th rib; LMY: lean meat yield; SF: shear force; CC: carcass conformation; IMFP: intramuscular fat percentage.

^5^ References reported indicating QTLs or SNPs.

## Discussion

In this study, the LL muscle of Holstein steers and bulls were biochemically characterized, and the transcriptomes of the LL muscle were profiled. The results showed that castration improved beef quality yet reduced the meat yield of Holstein bulls. We obtained a total of 56 DEGs, with 37 being upregulated and 19 being downregulated. Among them, seven promising candidate genes which were associated with beef quality upon castration were identified upon our integrated analysis, which included GO analysis, KEGG pathways enrichment analysis and screening with reported QTLs and SNPs. The seven genes included both muscle structural proteins coding genes (*MYH1*, *MYH4*, *MYH10*) and functional proteins coding genes (*GADL1*, *CYP2R1*, *EEPD1*, *SHISA3*). With the exceptions of *MYH1* and *MYH4*, the remaining five genes are novel reported genes related to beef quality traits [34, 35]. Our findings generate a complete data set of genes in the LL muscle that are affected by castration.

*MYH1*, *MYH4* and *MYH10* are different isoforms of the myosin heavy chain gene family (MYH), and are responsible for the diversities of skeletal muscle fiber types. Myosin fibers are mainly composed of four types, namely class I, class IIA, class IIX and class IIB, which correlate to the muscle morphologies and biochemical characteristics, ultimately impacting meat quality traits [36]. *MYH1* encodes the motor domain of class 2X isoform, while *MYH4* and *MYH10* both encode the motor domain of class 2B isoform. A previous study regarding the meat qualities of pork revealed that class 2B and 2X are the most abundant fibers representing the muscle mass, and affect meat quality based on a positive correlation with pH decrease ratio and marbling score [37]. Interestingly, our QTL-SNP screening for beef qualify related genes revealed that *MYH1*, *MYH4* and *MYH10* were all located within QTL regions associated with IMF, and close to SNPs associated with fatty acids composition and carcass conformation, thus indicating that these myosin heavy chain genes mainly affected beef quality through meat composition (Table 4). Proteomic study revealed that *MYH1* was associated with superior beef tenderness [38]. A microarray analysis at the transcriptomic level showed that *MYH1* was related to the shear force of beef muscle [34]. Similarly, our RNA-seq analysis showed that *MYH1* expression was significantly increased in the LL of steers with better beef quality, coordinating with its identified role in improving beef quality. Whole genome resequencing demonstrated that *MYH4*, as a positive marker of meat quality improvement, was related with the skeletal muscle development and muscle fiber type for red Angus cattle [35]. Our results revealed that the *MYH4* mRNA level significantly increased in the steers in comparison to the bulls, indicating *MYH4* may play a similar role in positively affecting beef quality traits. Although beef quality of the steers was significantly higher than that of the bulls, but the *MYH10* level was relatively lower in the steers’ muscle. Hence, *MYH10* showed negative correlations with beef quality traits. This is also the first report that myosin heave chain gene *MYH10* is associated with beef quality traits. Coincidently, a transcriptome analysis of porcine *longissimus dorsi* muscle suggested that, compared to purebred Iberian pigs, the *MYH10* level was higher in crossbred Iberian pigs which contained lower IMF [39]. Therefore, both studies indicated that *MYH10* is negatively correlated with meat quality traits.

Glutamate decarboxylase, such as protein 1 (*GADL1*), encodes a protein that is specifically expressed in cattle muscle and functions in β-alanine and taurine production, and is ultimately involved in carnosine and pantothenate biosynthesis [40]. *GADL1* transcript level was upregulated in the steers, thus indicating its role in improving beef quality. Our beef quality related candidate genes screening revealed that *GADL1* is located within a QTL region related to lean meat yield, at a position close to significant SNPs with carcass conformation and LL muscle area (Table 4). It has been previously observed that carnosine and taurine can promote the stability of IMF lipids and beef color [41]. In addition, nutrition studies regarding pigs and geese revealed that supplementing with pantothenic acid improves carcass characteristics, lipid metabolism and meat quality, thus indicating that pantothenic acid may play a similar nutrient role in cattle [42,43]. Our data suggested that the promising functions of carnosine, taurine and pantothenic acid in improving meat quality may be related to the gene *GADL1*. Overall, we proposed that *GADL1* is likely associated with lipid metabolism and carcass, but this requires further investigation.

Cytochrome P450 family 2 subfamily R member 1 (*CYP2R1*) encodes an enzyme of the cytochrome P450 superfamily. *CYP2R1* is highly conserved from zebrafish, chicken to mammals. Its human homolog catalyzes steroid biosynthesis, thereby implicating a similar role in cattle [44]. Since steroid is a precursor of androgen, the decrease of *CYP2R1* mRNA level in steers may be straightly explained by lower androgen level after castration. At the same time, it was previously reported that vitamin D_3_ supplementation improved beef tenderness in steers [45]. Coincidingly, the results of our QTL-SNP screening suggested that the *CYP2R1* gene loci are close to an SNP associated with IMF percentage which is responsible for tenderness (Table 4). The KEGG analysis suggested that CYP2R1 catalyzed vitamin D_3_, so as to produce calcidiol. The decrease of the *CYP2R1* expression level in the LL of steers may cause the accumulation of vitamin D_3_, which further improved the beef tenderness. Therefore, we propose that *CYP2R1* may be an important candidate gene that is related to beef tenderness, mainly through its involvement in vitamin D_3_ accumulation.

Shisa family member 3 (*SHISA3*) encodes a single-transmembrane protein of the Shisa family. Our QTL-SNP screening showed that *SHISA3* is associated with beef quality traits such as shear force and IMF percentage, indicating the function of SHISA3 might be related with adipose deposition. Previous study revealed that SHISA3 plays a role as a tumor suppressor in lung cancer, by inhibiting the Wnt/β-catenin signaling pathway in lung adenocarcinoma cells [46]. Coincidently, Wnt/β-catenin signaling pathway was reported as potent inhibitor of adipogenesis [47]. A study on Korean cattle indicated that Wnt/β-catenin signaling pathway was downregulated following castration, which negatively corelates with increased IMF in *longissimus dorsi* muscle [48]. If SHISA3 regulates Wnt/β-catenin signaling pathway in muscle cells in the same way as lung adenocarcinoma cells, expression of SHISA3 should have shown positive correlations with beef quality traits. However, our study revealed that the transcription level of *SHISA3* was downregulated in the steers compared to the bulls, showing a negative correlation with higher IMF content in steers. Since the function of SHISA3 in cattle has not been studied yet, it is worth to investigate the mechanism how SHISA3 impacts adipogenesis or adipose deposition in future studies. Regulation of SHISA3 on Wnt/β-catenin signaling pathway in LL muscle will be of particular interest in order to better explain our findings.

The endonuclease/exonuclease/phosphatase family domain containing 1 (*EEPD1*) encoded gene that mainly plays a role in repairing stressed replication forks during DNA damage in humans [49]. *EEPD1* in cattle has not been studied extensively, but a transcriptome analysis of chicken adipose tissue revealed that a decrease of *EEPD1* correlated with both fasting and insulin-neutralization, thus indicating its negative regulation in fatty acids synthesis [50]. Our QTL-SNP screening of DEG-related beef quality suggested that EEPD1 was located within a QTL region associated with carcass weight and fat thickness at the 12th rib, and near SNPs impact carcass conformation and marbling score (Table 4). Therefore, *EEPD1* may affect lipid metabolism or fat deposition. Opposite to the results of the chicken study, in our study *EEPD1* was upregulated in the LL of steers, which contains significantly higher IMF, indicating that it may play a positive role in fat deposition in cattle muscle. In addition, a study by Nelson *et al*. on human and murine macrophages proved that *EEPD1* is regulated by LXR, which is a responsive receptor of high cellular sterol load, while *EEPD1* contributes to the efflux of excess cellular cholesterol [51]. These findings indicate that EEPD1 may play a potential role as an androgen sensor in muscle. Subsequently, in order to detect whether *EEPD1* could be a potential candidate gene associated with reproduction traits, we compared the physical location of the *EEPD1* gene with the reported QTL regions and SNPs associated with cattle reproduction traits. It was clear that the physical location of the *EEPD1* dropped into the QTL regions related with scrotal circumference and gestation length, and was very close to the SNPs associated with poor sperm motility and cow conception rate (S8 Table). Although the indicated QTLs and SNPs were discovered in reproductive organs, traits such as scrotal circumference, sperm motility and gestation length are all corelated with sex hormone levels directly or indirectly. From this prospective, and linking the research by the Nelson Group, we cannot exclude the possibility that the upregulation of *EEPD1* in LL muscle of steers is a response to higher cellular steroid level induced by castration. Therefore, EEPD1 became a strong candidate gene with overlapping functions of sensing castration effects, subsequently improving beef quality.

Overall, our study reported 56 DEGs in the LL of Holstein bulls and steers. Our QTL-SNP screening analysis revealed seven candidate genes potentially associated with beef quality improvement caused by castration, including muscle structural proteins coding genes (*MYH1*, *MYH4*, *MYH10*) and functional proteins coding genes (*GADL1*, *CYP2R1*, *EEPD1*, *SHISA3*). Among them, *GADL1*, *CYP2R1*, *EEPD1*, *SHISA3* and *MYH10* were novel candidate genes associated with beef quality traits. *EEPD1* is associated with both beef quality and reproduction traits, providing important insight as to how castration affected the beef quality at the gene level. Our study confirmed that the reported positive markers of meat quality traits *MYH4* and *MYH1* are associated with the beef quality improvement following castration. We also identified another myosin heavy chain family gene *MYH10*, which is negatively associated with beef quality traits. Hence, these data demonstrated that the myosin heavy chain family plays an important role in regulating beef quality. Due to the fact that our QTL-SNP screening was biased on screening genes associated with meat quality traits, it is possible that the DEGs related only to the sex hormone responses, but not meat quality traits, were omitted. The overall analysis indicated castration affected gene expression in LL muscle, resulting in beef quality improvement in Holstein steers.

## Supporting information

S1 Table Composition and nutrient levels of basal diets (DM^1^ basis). (DOCX)

S2 Table Primers for qRT-PCR of 9 random selected differentially expressed genes. (DOCX)

S3 Table The summary of RNA-seq reads generated from bulls and steers. (DOCX)

S4 Table The data of RNA-seq Reads alignments to *Bos taurus* genome. (DOCX)

S5 Table The statistical analysis of gene expression level in *longissimus lumborum* from bulls and steers. (DOCX)

S6 Table Screening of DEGs between LL of steers and bulls by distances with reported QTLs associated with meat quality traits. (DOCX)

S7 Table Screening of DEGs between LL of steers and bulls by distances with reported SNPs associated with meat quality traits. (DOCX)

S8 Table Analysis of EEPD1 with reported QTLs and SNPs associated with reproduction traits. (DOCX)
