# Supplementary material for: Transcriptome profiling of longissimus lumborum in Holstein bulls and steers with different beef qualities
Source: PLoS One. 2020 Jun 25;15(6):e0235218. doi: 10.1371/journal.pone.0235218 (PMC7316285; doi:10.1371/journal.pone.0235218)
Supplement: S1 Table — (DOCX) [file pone.0235218.s001.docx]

**S1 Table. Composition and nutrient levels of basal diets (DM^1^ basis).**

| **Items** | **Live weight** | | | | | | | | | |
| --- | --- | --- | --- | --- | --- | --- | --- | --- | --- | --- |
|  | **270~320 kg** | **321~370 kg** | **371~420 kg** | **421~470 kg** | **471~520 kg** | **521kg~570kg** | **571kg~620kg** | **621kg~670kg** | **671kg~720kg** | **721kg~770kg** |
| **Corn (%)** | 30.23 | 37.22 | 41.88 | 48.00 | 48.76 | 50.14 | 50.86 | 51.82 | 52.69 | 60.20 |
| **Soybean meal (%)** | 5.15 | 2.13 | 3.86 | 1.05 | 1.02 | 0.00 | 0.00 | 0.00 | 0.00 | 3.60 |
| **Cottonseed meal (%)** | 10.14 | 8.68 | 3.60 | 6.15 | 6.12 | 6.00 | 6.00 | 6.02 | 6.00 | 0.00 |
| **DDGS^2^ (%)** | 8.90 | 6.76 | 8.85 | 6.56 | 6.69 | 7.30 | 7.35 | 7.32 | 7.35 | 7.00 |
| **Wheat bran (%)** | 0.00 | 3.40 | 0.00 | 0.00 | 0.00 | 0.00 | 0.00 | 0.00 | 0.00 | 0.90 |
| **Limestone (%)** | 1.21 | 0.44 | 0.44 | 0.78 | 0.78 | 0.99 | 1.07 | 1.09 | 1.12 | 1.25 |
| **Premix^3^ (%)** | 0.34 | 0.34 | 0.34 | 0.32 | 0.39 | 0.33 | 0.40 | 0.41 | 0.41 | 0.40 |
| **Baking soda%)** | 0.69 | 0.69 | 0.69 | 0.82 | 0.85 | 0.84 | 0.92 | 0.93 | 1.02 | 1.25 |
| **NaCl (%)** | 0.34 | 0.34 | 0.34 | 0.32 | 0.39 | 0.40 | 0.40 | 0.41 | 0.41 | 0.40 |
| **Corn stalk silage (%)** | 43.00 | 40.00 | 40.00 | 36.00 | 35.00 | 34.00 | 33.00 | 32.00 | 31.00 | 25.00 |
| **Total (%)** | 100.00 | 100.00 | 100.00 | 100.00 | 100.00 | 100 | 100 | 100 | 100 | 100 |
| **Nutrient levels** |  |  |  |  |  |  |  |  |  |  |
| **NEmf^4^ (MJ/kg)** | 6.69 | 6.87 | 6.92 | 7.08 | 7.11 | 7.11 | 7.13 | 7.17 | 7.21 | 7.53 |
| **Crude protein (%)** | 15.57 | 14.04 | 13.00 | 12.50 | 12.50 | 12.26 | 12.26 | 12.26 | 12.26 | 11.41 |
| **Calcium (%)** | 0.65 | 0.52 | 0.56 | 0.59 | 0.59 | 0.63 | 0.66 | 0.66 | 0.63 | 0.77 |
| **Phosphorus (%)** | 0.33 | 0.46 | 0.40 | 0.39 | 0.39 | 0.39 | 0.39 | 0.39 | 0.39 | 0.35 |
| **NDF^5^ (%)** | 40.71 | 39.07 | 37.40 | 35.03 | 29.16 | 28.50 | 28.67 | 28.09 | 27.49 | 25.03 |
| **ADF^6^ (%)** | 18.78 | 17.63 | 16.60 | 15.58 | 16.01 | 15.58 | 12.35 | 12.05 | 11.76 | 10.43 |

Note: ^1^DM: dry matter

^2^DDGS: Distiller's Dried Grain with Soluble

^3^Premix is provided for per kg ration: vitamin A 4300IU, vitamin D3 650IU, vitamin E 25IU, copper 8mg, iron 70mg, manganese 40mg, zinc 60mg, iodine 0.5ng, selenium 0.1mg, cobalt 0.4mg.

^4^NEmf: combined net energy, calculated from Japanese Feeding Standard for Beef Cattle.

^5^NDF: neutral detergent fiber

^6^ADF: acid detergent fiber
