# Supplementary material for: Transcriptome profiling of longissimus lumborum in Holstein bulls and steers with different beef qualities
Source: PLoS One. 2020 Jun 25;15(6):e0235218. doi: 10.1371/journal.pone.0235218 (PMC7316285; doi:10.1371/journal.pone.0235218)
Supplement: S2 Table — (DOCX) [file pone.0235218.s002.docx]

**S2 Table. Primers for qRT-PCR of 9 random selected differentially expressed genes.**

| NCBI reference sequence | Gene Name | Forward Primer | Reverse Primer | Product Size |
| --- | --- | --- | --- | --- |
| [NM_001105327.2](https://www.ncbi.nlm.nih.gov/entrez/viewer.fcgi?db=nucleotide&id=402744401) | IGFBP5 | GCAAGCCAAGATCGAAAGAGA | TCTGCGGTCCTTCTTCACAG | 148 |
| [NM_001191279.2](https://www.ncbi.nlm.nih.gov/entrez/viewer.fcgi?db=nucleotide&id=433660839) | PLCL1 | AGTATTGAAGGGCCAGGGAGA | CACTGCTGGGGGCTTTACTA | 116 |
| [NM_001077836.1](https://www.ncbi.nlm.nih.gov/entrez/viewer.fcgi?db=nucleotide&id=118150785) | SLC6A1 | ACAGCCAGTTCTGTACAGTGG | GATGTTGGACAGGCCGATCA | 134 |
| [NM_001034600.2](https://www.ncbi.nlm.nih.gov/entrez/viewer.fcgi?db=nucleotide&id=402745287) | BDH1 | TGGCTGTTTGATGAAGGACAAA | TCGCTTTTGCAGACGTTGAG | 102 |
| [XM_002695806.4](https://www.ncbi.nlm.nih.gov/entrez/viewer.fcgi?db=nucleotide&id=982957405) | MYH4 | GGTCCAAGTGCTGAAGAGGGT | CCTCAGGTTGGTCATCAGCTT | 120 |
| [NM_001076814.1](https://www.ncbi.nlm.nih.gov/entrez/viewer.fcgi?db=nucleotide&id=116003846) | ME2 | AGAACAAACAAGGGGATGGC | TGGAACCGTAAGGCTTGAAT | 110 |
| [NM_001015672.2](https://www.ncbi.nlm.nih.gov/entrez/viewer.fcgi?db=nucleotide&id=75832110) | RETREG1 | CCGATGTGTCTGAGGTCTCC | CTAGGTCGGTCAAGGTCATCAG | 100 |
| [NM_001076843.1](https://www.ncbi.nlm.nih.gov/entrez/viewer.fcgi?db=nucleotide&id=116003902) | SLC30A3 | GCACACCTGGCCATTGACTC | GCAGGGTACAGCTGGAGAAT | 100 |
