# Supplementary material for: Transcriptome profiling of longissimus lumborum in Holstein bulls and steers with different beef qualities
Source: PLoS One. 2020 Jun 25;15(6):e0235218. doi: 10.1371/journal.pone.0235218 (PMC7316285; doi:10.1371/journal.pone.0235218)
Supplement: S3 Table — (DOCX) [file pone.0235218.s003.docx]

**S3 Table. The summary of RNA-seq reads generated from bulls and steers.**

| Sample name | Raw reads | Clean reads | Clean bases | Error rate (%) | Q20(%) | Q30(%) | GC content(%) |
| --- | --- | --- | --- | --- | --- | --- | --- |
| B_LL1 | 53583218 | 47803514 | 7.17G | 0.03 | 94.64 | 87.96 | 53.65 |
| B_LL2 | 56513280 | 53912628 | 8.09G | 0.02 | 95.84 | 89.53 | 54.00 |
| B_LL3 | 56979280 | 54416018 | 8.16G | 0.02 | 95.85 | 89.54 | 53.98 |
| S_LL1 | 57562944 | 51661120 | 7.75G | 0.03 | 94.73 | 88.11 | 53.39 |
| S_LL2 | 48488012 | 43339374 | 6.5G | 0.03 | 94.73 | 88.13 | 53.88 |
| S_LL3 | 57163642 | 54434094 | 8.17G | 0.02 | 95.96 | 89.78 | 54.66 |

Note: In sample names, B represents bulls, while S stands for steers. LL means *longissimus lumborum,* and numbers 1/2/3 indicate three replicates in responding group.

Q20: **the proportion of bases with a** phred base quality score greater than 20; i.e., the proportion of read bases whose error rate is less than 1%.

Q30: **the proportion of bases with a** phred base quality score greater than 30; i.e., the proportion of read bases whose error rate is less than 0.1%.
