# Supplementary material for: Transcriptome profiling of longissimus lumborum in Holstein bulls and steers with different beef qualities
Source: PLoS One. 2020 Jun 25;15(6):e0235218. doi: 10.1371/journal.pone.0235218 (PMC7316285; doi:10.1371/journal.pone.0235218)
Supplement: S4 Table — (DOCX) [file pone.0235218.s004.docx]

**S4 Table. The data of RNA-seq Reads alignments to *Bos taurus* genome.**

| Sample_name | B_LL1 | B_LL2 | B_LL3 | S_LL1 | S_LL2 | S_LL3 |
| --- | --- | --- | --- | --- | --- | --- |
| Total reads | 47803514 | 53912628 | 54416018 | 51661120 | 43339374 | 54434094 |
| Total mapped | 43049008  (90.05%) | 49942574  (92.64%) | 50391818  (92.6%) | 46469251  (89.95%) | 39040459  (90.08%) | 50182901  (92.19%) |
| Multiple mapped | 1113629  (2.33%) | 1351199  (2.51%) | 1636707  (3.01%) | 1494765  (2.89%) | 1385121  (3.2%) | 1993803  (3.66%) |
| Uniquely mapped | 41935379  (87.72%) | 48591375  (90.13%) | 48755111  (89.6%) | 44974486  (87.06%) | 37655338  (86.88%) | 48189098  (88.53%) |
| Reads map to '+' | 20932778  (43.79%) | 24258300  (45%) | 24346817  (44.74%) | 22459142  (43.47%) | 18815291  (43.41%) | 24113519  (44.3%) |
| Reads map to '-' | 21002601  (43.94%) | 24333075  (45.13%) | 24408294  (44.85%) | 22515344  (43.58%) | 18840047  (43.47%) | 24075579  (44.23%) |
| Non-splice reads | 23569173  (49.3%) | 27282887  (50.61%) | 25605360  (47.05%) | 24009173  (46.47%) | 19105282  (44.08%) | 24354543  (44.74%) |
| Splice reads | 18366206  (38.42%) | 21308488  (39.52%) | 23149751  (42.54%) | 20965313  (40.58%) | 18550056  (42.8%) | 23834555  (43.79%) |

Note: *Bos taurus* genome **UMD3.1 (**[ftp://ftp.ensembl.org/pub/release-88/fasta/bos_taurus/dna/](file:///F:\pub\release-88\fasta\bos_taurus\dna)) was used for the alignment.
