# Supplementary material for: Transcriptome profiling of longissimus lumborum in Holstein bulls and steers with different beef qualities
Source: PLoS One. 2020 Jun 25;15(6):e0235218. doi: 10.1371/journal.pone.0235218 (PMC7316285; doi:10.1371/journal.pone.0235218)
Supplement: S5 Table — (DOCX) [file pone.0235218.s005.docx]

**S5 Table. The statistical analysis of gene expression level in *longissimus lumborum* from bulls and steers.**

| FPKM Interval | B_LL1 | B_LL2 | B_LL3 | S_LL1 | S_LL2 | S_LL3 |
| --- | --- | --- | --- | --- | --- | --- |
| 0~1 | 15489  (54.25%) | 15872  (55.59%) | 16163  (56.61%) | 15984  (55.98%) | 15957  (55.89%) | 16387  (57.39%) |
| 1~3 | 3174  (11.12%) | 3115  (10.91%) | 3059  (10.71%) | 3098  (10.85%) | 3116  (10.91%) | 3079  (10.78%) |
| 3~15 | 5799  (20.31%) | 5631  (19.72%) | 5338  (18.70%) | 5575  (19.53%) | 5577  (19.53%) | 5368  (18.80%) |
| 15~60 | 2855  (10.00%) | 2775  (9.72%) | 2762  (9.67%) | 2700  (9.46%) | 2706  (9.48%) | 2584  (9.05%) |
| >60 | 1235  (4.33%) | 1159  (4.06%) | 1230  (4.31%) | 1195  (4.19%) | 1196  (4.19%) | 1134  (3.97%) |
