# Supplementary material for: Transcriptome profiling of longissimus lumborum in Holstein bulls and steers with different beef qualities
Source: PLoS One. 2020 Jun 25;15(6):e0235218. doi: 10.1371/journal.pone.0235218 (PMC7316285; doi:10.1371/journal.pone.0235218)
Supplement: S6 Table — (DOCX) [file pone.0235218.s006.docx]

**S6 Table. Screening of DEGs between LL of steers and bulls by distances with reported QTLs associated with meat quality traits.**

| Gene Symbol | Chr^1^ | Position  (bp)^2^ | Position  (cM) | Distance to QTL peak  (cM) | QTL ID | CI  (cM) | Peak location (cM) | Traits^4^ | References |
| --- | --- | --- | --- | --- | --- | --- | --- | --- | --- |
| *EEPD1* | 4 | 61354648-  61476979 | 60.74 | 0.6 | 20355 | 60.14-60.14 | 60.14 | CW | Nalaila et al., *J. Anim. Breed. Genet.* **2012**, 129, 107-119 |
|  |  |  |  | 0.6 | 20368 | 60.14-60.14 | 60.14 | FT12R | Nalaila et al., *J. Anim. Breed. Genet.* **2012**, 129, 107-119 |
| *MYH10* | 19 | 28680825-  28800880 | 49.04 | -22.3 | 22873 | 53.65-87.68 | 71.37 | IMF | Peters et al., *J. Anim. Sci.* **2012***,* 90, 3398-3409 |
|  |  |  |  | -2.6 | 22872 | 53.24-53.77 | 51.62 | IMF | Peters et al., *J. Anim. Sci.* **2012**, 90, 3398-3409 |
| *CYP2R1* | 15 | 38420926-  38445583 | 46.87 | -6.7 | 24706 | 52.94-54.27 | 53.60 | LDMA | Saatchi et al., *BMC Genomics* **2014**, 15, 442-2164-15-442 |
| *GADL1* | 22 | 5258463-  5452369 | 6.31 | -2.0 | 37164 | 8.32-8.32 | 8.32 | LMY | Doran et al., *BMC Genomics* **2014**, 15, 837-2164-15-837 |
| *MYH1* | 19 | 30110728-  30134757 | 51.49 | -19.9 | 22873 | 53.65-87.68 | 71.37 | IMF | Peters et al., *J. Anim. Sci.* **2012**, 90, 3398-3409 |
| *SHISA3* | 6 | 62877287-  62880849 | 70.42 | -0.8 | 20764 | 23.62-118.80 | 71.21 | SF | McClure et al., *Anim. Genet.* **2012**, 43, 662-673 |
| *MYH4* | 19 | 30080604-  30103436 | 51.44 | -19.9 | 22873 | 53.65-87.68 | 71.37 | IMF | Peters et al., *J. Anim. Sci.* **2012**, 90, 3398-3409 |
| *KIAA1211* | 6 | 73368358-  73396166 | 82.17 | 11.0 | 20764 | 23.62-118.80 | 71.21 | SF | McClure et al., *Anim. Genet.* **2012**, 43, 662-673 |
| *CST6* | 29 | 44770865-  44771529 | 60.44 | 13.8 | 19759 | 25.70-67.58 | 46.64 | MAC | Saatchi et al., *BMC Genomics* **2013**, 14, 730-2164-14-730 |

^1^Chromosome in *B. taurus.*

^2^Gene position on the UMD3.1.1 bovine genome assembly.

^3^QTL information retrieved on the Animal Quantitative Trait Loci (QTL) Database (Animal QTLdb) (<https://www.animalgenome.org/cgi-bin/QTLdb/index>).

^4^CW: carcass weight; IMF: intramuscular fat; FT12R: fat thickness at the 12th rib; LDMA: *Longissimus dorsi* muscle area(cm^2^); LMY: lean meat yield; SF: shear force; MAC: margaric acid content.
