# Supplementary material for: Transcriptome profiling of longissimus lumborum in Holstein bulls and steers with different beef qualities
Source: PLoS One. 2020 Jun 25;15(6):e0235218. doi: 10.1371/journal.pone.0235218 (PMC7316285; doi:10.1371/journal.pone.0235218)
Supplement: S7 Table — (DOCX) [file pone.0235218.s007.docx]

**S7 Table. Screening of DEGs between LL of steers and bulls by distances with reported SNPs associated with meat quality traits.**

| Gene Symbol | Chr^1^ | Gene Position^2^  (bp) | Distance to SNP  (Mb) | SNP Name | SNP Position^3^  (bp) | Traits^4^ | Raw  P-Value | Reference |
| --- | --- | --- | --- | --- | --- | --- | --- | --- |
| *GADL1* | 22 | 5258463-5452369 | 0.06 | ARS-BFGL-NGS-27027 | 5202348 | CC | 0.044 | Doran et al., *BMC Genomics* **2014,** *15,* 837-2164-15-837 |
| *CYP2R1* | 15 | 38420926-38445583 | 2.12 | ARS-BFGL-NGS-117790 | 40567835 | CC | 0.031 | Doran et al., *BMC Genomics* **2014,** *15,* 837-2164-15-837 |
| *MYH1* | 19 | 30110728-30134757 | 2.58 | UA-IFASA-9813 | 32716622 | CW | 0.047 | Doran et al., *BMC Genomics* **2014,** *15,* 837-2164-15-837 |
|  |  |  | 2.25 | ARS-BFGL-NGS-88422 | 27858990 | CC | 0.050 | Doran et al., *BMC Genomics* **2014,** *15,* 837-2164-15-837 |
| *EEPD1* | 4 | 61354648-61476979 | 1.84 | Hapmap42645-BTA-70875 | 63317686 | CC | 0.012 | Doran et al., *BMC Genomics* **2014,** *15,* 837-2164-15-837 |
| *MYH10* | 19 | 28680825-28800880 | 3.92 | UA-IFASA-9813 | 32716622 | CW | 0.047 | Doran et al., *BMC Genomics* **2014,** *15,* 837-2164-15-837 |
|  |  |  | 0.82 | ARS-BFGL-NGS-88422 | 27858990 | CC | 0.050 | Doran et al., *BMC Genomics* **2014,** *15,* 837-2164-15-837 |
| *SHISA3* | 6 | 62877287-62880849 | 1.13 | BTA-76543-no-rs | 61750466 | IMFP | 3.00E-04 | Lee et al., *Livestock Science* **2014,** *165,* 27-32 |
| *MYH4* | 19 | 30080604-30103436 | 2.61 | UA-IFASA-9813 | 32716622 | CW | 0.047 | Doran et al., *BMC Genomics* **2014,** *15,* 837-2164-15-837 |
|  |  |  | 2.22 | ARS-BFGL-NGS-88422 | 27858990 | CC | 0.050 | Doran et al., *BMC Genomics* **2014,** *15,* 837-2164-15-837 |
| *KIAA1211* | 6 | 73368358-73396166 | 1.49 | BTA-76627-no-rs | 71877286 | CC | 0.028 | Doran et al., *BMC Genomics* **2014,** *15,* 837-2164-15-837 |
| *CEBPD* | 14 | 20738814-20740407 | 1.52 | ARS-BFGL-NGS-104268 | 22260372 | IMFP, RFC | 0.004 | Bolormaa et al., *J. Anim. Sci.* **2011,** *89,* 2297-2309 |
|  |  |  | 3.64 | BTB-01143580 | 24383627 | CW | 6.35E-11 | Lee et al., *PLoS One* **2013,** *8,* e74677 |
|  |  |  | 4.38 | ARS-BFGL-NGS-12947 | 25116073 | CC | 0.048 | Doran et al., *BMC Genomics* **2014,** *15,* 837-2164-15-837 |
| *ZSWIM4* | 7 | 12959854-12977638 | 3.69 | ARS-BFGL-NGS-107510 | 16671812 | CC | 0.046 | Doran et al., *BMC Genomics* **2014,** *15,* 837-2164-15-837 |
|  |  |  | 1.17 | AX-26832968 | 14142752 | MA | 0.006 | Hyeong et al., *Asian-Australas J Anim Sci* **2014,** *27(9),* 1219-1227 |
| *PADI2* | 2 | 136049644-136103406 | 1.70 | BTB-00107514 | 137799962 | CC | 0.043 | Doran et al., *BMC Genomics* **2014,** *15,* 837-2164-15-837 |
| *ROCK2* | 11 | 86501577-86583652 | 4.59 | ARS-BFGL-NGS-106479 | 81908400 | IMFP | 0.036 | Bolormaa et al., *J. Anim. Sci.* **2011,** *89,* 2297-2309 |
|  |  |  | 0.02 | ARS-BFGL-NGS-34903 | 86478142 | CC | 0.016 | Doran et al., *BMC Genomics* **2014,** *15,* 837-2164-15-837 |
| *IGFBP5* | 2 | 105378991-105397646 | 3.56 | ARS-BFGL-NGS-99780 | 101815166 | CC | 0.035 | Doran et al., *BMC Genomics* **2014,** *15,* 837-2164-15-837 |
|  |  |  | 4.56 | Hapmap49626-BTA-48806 | 109956378 | IMFP | 4.00E-04 | Lee et al., *Livestock Science* **2014,** *165,* 27-32 |
| *PLCL1* | 2 | 86718341-87086748 | 1.98 | Hapmap34569-BES3_Contig278_695 | 89062659 | CC | 0.032 | Doran et al., *BMC Genomics* **2014,** *15,* 837-2164-15-837 |
|  |  |  | 3.11 | AX-22345506 | 90197319 | MT | 0.020 | Hyeong et al., *Asian-Australas J Anim Sci* **2014,** *27(9),* 1219-1227 |
| *CDKN1A* | 23 | 10560499-10568780 | 4.33 | INRA-443 | 6228806 | IMFP | 0.009 | Bolormaa et al., *J. Anim. Sci.* **2011,** *89,* 2297-2309 |
|  |  |  | 1.77 | ARS-USMARC-Parent-AY929334-no-rs | 8790334 | CC | 0.032 | Doran et al., *BMC Genomics* **2014,** *15,* 837-2164-15-837 |
| *FBXL22* | 10 | 46461587-46466076 | 0.13 | ARS-BFGL-BAC-13522 | 46596553 | CC | 0.020 | Doran et al., *BMC Genomics* **2014,** *15,* 837-2164-15-837 |
| *GANC* | 10 | 37754445-37817713 | 0.65 | Hapmap55139-rs29022040 | 38463822 | CC | 0.007 | Doran et al., *BMC Genomics* **2014,** *15,* 837-2164-15-837 |
|  |  |  | 2.21 | AX-18755438 | 35548753 | MA | 0.048 | Hyeong et al., *Asian-Australas J Anim Sci* **2014,** *27(9),* 1219-1227 |
| *NPTX1* | 19 | 52679104-52685498 | 3.86 | ARS-BFGL-NGS-414 | 48823524 | CF | 0.047 | Doran et al., *BMC Genomics* **2014,** *15,* 837-2164-15-837 |
|  |  |  | 3.06 | ARS-BFGL-NGS-78203 | 49617253 | CC | 0.034 | Doran et al., *BMC Genomics* **2014,** *15,* 837-2164-15-837 |
| *GPA33* | 3 | 1726504-1779667 | 2.61 | ARS-BFGL-NGS-35211 | 4392929 | IMFP | 8.00E-04 | Lee et al., *Livestock Science* **2014,** *165,* 27-32 |
| *STBD1* | 6 | 92967767-92971371 | 0.99 | ARS-BFGL-NGS-60568 | 93962054 | CC | 0.031 | Doran et al., *BMC Genomics* **2014,** *15,* 837-2164-15-837 |
| *SLC30A3* | 11 | 72364682-72373190 | 1.34 | Hapmap36263-SCAFFOLD265651_7554 | 71024437 | CC | 0.022 | Doran et al., *BMC Genomics* **2014,** *15,* 837-2164-15-837 |
|  |  |  | 0.63 | AX-19331523 | 73007955 | MT | 0.030 | Hyeong et al., *Asian-Australas J Anim Sci* **2014,** *27(9),* 1219-1227 |
|  |  |  | 2.21 | ARS-BFGL-NGS-37893 | 74578375 | IMFP | 5.00E-04 | Lee et al., *Livestock Science* **2014,** *165,* 27-32 |
| *AMPD1* | 3 | 28756908-28768496 | 4.73 | ARS-BFGL-NGS-119919 | 33497831 | CC | 0.036 | Doran et al., *BMC Genomics* **2014,** *15,* 837-2164-15-837 |
|  |  |  | 1.78 | rs110049045 | 26975095 | PAC; OAC | 1.37E-04;  7.04E-05 | Cesar et al., *BMC Genet.* **2014,** *15,* 39-2156-15-39 |
| *MSTN* | 2 | 6213566-6220196 | 0.08 | AX-22028107 | 6300508 | MA | 0.018 | Hyeong et al., *Asian-Australas J Anim Sci* **2014,** *27(9),* 1219-1227 |
| *CST6* | 29 | 44766936-44768173 | 0.21 | ARS-BFGL-NGS-37441 | 44977945 | MTLM | 0.016 | Bolormaa et al., *J. Anim. Sci.* **2011,** *89,* 2297-2309 |
|  |  |  | 0.75 | ARS-BFGL-NGS-24243 | 45521523 | CC | 0.043 | Doran et al., *BMC Genomics* **2014,** *15,* 837-2164-15-837 |
| *GREB1* | 11 | 86199420-86268193 | 4.29 | ARS-BFGL-NGS-106479 | 81908400 | IMFP | 0.036 | Bolormaa et al., *J. Anim. Sci.* **2011,** *89,* 2297-2309 |
|  |  |  | 0.21 | ARS-BFGL-NGS-34903 | 86478142 | CC | 0.015 | Doran et al., *BMC Genomics* **2014,** *15,* 837-2164-15-837 |
| *ME2* | 24 | 50870262-50928290 | 3.75 | AX-23847051 | 54680656 | MP | 0.028 | Hyeong et al., *Asian-Australas J Anim Sci* **2014,** *27(9),* 1219-1227 |
| *RETREG1* | 20 | 56709603-56758641 | 0.06 | Hapmap40003-BTA-50839 | 56645366 | CC | 0.043 | Doran et al., *BMC Genomics* **2014,** *15,* 837-2164-15-837 |
| *GSTM1* | 3 | 33824401-33834874 | 0.33 | ARS-BFGL-NGS-119919 | 33497831 | CC | 0.036 | Doran et al., *BMC Genomics* **2014,** *15,* 837-2164-15-837 |
| *RAB11FIP3* | 25 | 404964-464143 | 1.81 | ARS-BFGL-NGS-79851 | 2278557 | CF | 0.047 | Doran et al., *BMC Genomics* **2014,** *15,* 837-2164-15-837 |
|  |  |  | 0.65 | ARS-BFGL-NGS-40627 | 1115387 | CC | 0.016 | Doran et al., *BMC Genomics* **2014,** *15,* 837-2164-15-837 |
|  |  |  | 0.40 | ARS-BFGL-NGS-14220 | 636 | PAC | 8.51E-07 | Ishii et al., *Anim. Sci. J.* **2013,** *84,* 675-682 |
| *PTER* | 13 | 31202455-31278293 | 0.41 | ARS-BFGL-NGS-30975 | 31689780 | CC | 0.023 | Doran et al., *BMC Genomics* **2014,** *15,* 837-2164-15-837 |
|  |  |  | 1.94 | rs41683753 | 33219105 | POAC, MS | N/A | Mokry et al., *BMC Genet.* **2013,** *14,* 47-2156-14-47 |
| *SETBP1* | 24 | 45025456-45141793 | 1.05 | ARS-BFGL-NGS-110400 | 43976477 | CC | 0.043 | Doran et al., *BMC Genomics* **2014,** *15,* 837-2164-15-837 |
| *ABHD2* | 21 | 20998719-21106763 | 3.25 | BTA-51614-no-rs | 17751537 | CC | 0.044 | Doran et al., *BMC Genomics* **2014,** *15,* 837-2164-15-837 |
| *OLFM1* | 11 | 106675880-106712955 | 2.06 | Hapmap52927-rs29019310 | 108775233 | CC | 0.006 | Doran et al., *BMC Genomics* **2014,** *15,* 837-2164-15-837 |
| *NEPN* | 9 | 33595874-33619430 | 3.11 | AX-27809598 | 36731857 | MT | 0.032 | Hyeong et al., *Asian-Australas J Anim Sci* **2014,** *27(9),* 1219-1227 |
| *AMPD1* | 3 | 28737677-28745359 | 4.75 | ARS-BFGL-NGS-119919 | 33497831 | CC | 0.036 | Doran et al., *BMC Genomics* **2014,** *15,* 837-2164-15-837 |
|  |  |  | 1.76 | rs110049045 | 26975095 | PAC; OAC | 1.37E-04;  7.04E-05 | Cesar et al., *BMC Genet.* **2014,** *15,* 39-2156-15-39 |
| *BDH1* | 1 | 72572941-72608810 | 0.55 | ARS-BFGL-NGS-45476 | 73154505 | CC | 0.046 | Doran et al., *BMC Genomics* **2014,** *15,* 837-2164-15-837 |

^1^Chromosome in *B. taurus.*

^2^Gene position on the UMD3.1.1 bovine genome assembly.

^3^SNP information retrieved from indicated references.

^4^CC: carcass conformation; IMFP: intramuscular fat percentage; CF: carcass fat; MS: marbling score; CW: carcass weight; OAC: oleic acid content; POAC: palmitoleic acid content; PAC: palmitic acid content; MT: meat tenderness; MP: meat palatability; MA: meat aroma; MTLM: meat tenderness measured as peak force to shear the LM ; RFC: rump fat measured at the sacro-iliac crest in the chiller.
