# Supplementary material for: Transcriptome profiling of longissimus lumborum in Holstein bulls and steers with different beef qualities
Source: PLoS One. 2020 Jun 25;15(6):e0235218. doi: 10.1371/journal.pone.0235218 (PMC7316285; doi:10.1371/journal.pone.0235218)
Supplement: S8 Table — (DOCX) [file pone.0235218.s008.docx]

**S8 Table.** Analysis of EEPD1 with reported QTLs and SNPs associated with reproduction traits.

| *Gene Information* | | | |  | *Reported QTLs/SNPs* | | | | |
| --- | --- | --- | --- | --- | --- | --- | --- | --- | --- |
| Gene Symbol | **Chr^1^** | **Position（bp)^2^** | **Position**  **(cM)** | **Distance to peak (cM)** | **QTL ID^3^** | **CI (cM)** | **Peak location (cM)** | **Trait^4^** | **Reference^5^** |
| EEPD1 | 4 | 61354648-61476979 | 60.74 | -38.96 | 10718 | 87.33-107.1 | 99.7 | SCRCIR | McClure et al., *Anim Genet,* **2010**, *41 (6)*, 597-607 |
|  |  |  |  | -5.08 | 4621 | 102.1-124.8 | 65.82 | GLENGTH | Schrooten et al., *J. Dairy Sci.*, **2000**, *83 (4)*, 795-806 |
|  |  |  |  | **Distance to SNP (cM)** | **SNP Name (bp)^6^** | **SNP Position (bp)** | **Raw P value** | **Traits^4^** | **Reference^5^** |
|  |  |  |  | 7.93 | rs43399120 | 53420825 | 5.26E-05 | PSMOT | Hering et al., *Anim. Reprod. Sci.* **2014,** *146,* 89-97 |
|  |  |  |  | -6.01 | ARS-BFGL-NGS-27521 | 67489709 | 4.21E-04 | CCR | Parker Gaddis et al., *J. Dairy Sci.*, **2016**, *99(8)*, 6420–6435 |

^1^Chromosome in *B. taurus.*

^2^Gene position on the UMD3.1.1 bovine genome assembly.

^3^QTL information retrieved on the Animal Quantitative Trait Loci (QTL) Database (Animal QTLdb) (<https://www.animalgenome.org/cgi-bin/QTLdb/index>).

^4^SCRCIR: Scrotal circumference; GLENGTH: Gestation length; PSMOT: poor sperm motility; CCR: Cow conception rate.

^5^References reported indicated QTLs or SNPs.

^6^SNP information retrieved from indicated references.
